# Rapid Neurocognitive Deterioration and Mortality in a Healthcare Professional With Spongiform Encephalopathy: Implications for Neurologic and End-of-Life Care

**DOI:** 10.7759/cureus.14277

**Published:** 2021-04-03

**Authors:** Michael W Kortz, Brian M Kongs, Lauren E Middleton

**Affiliations:** 1 Neurosurgery, College of Osteopathic Medicine, Kansas City University, Kansas City, USA; 2 Neurology, College of Osteopathic Medicine, Kansas City University, Kansas City, USA; 3 Bioethics, Kansas City University, Kansas City, USA

**Keywords:** end of life and hospice care, prion diseases, spongiform encephalopathy, adult neurology, infectious disease, clinical case report, palliative and supportive care, ethics, multidisciplinary care, dissociation

## Abstract

Spongiform encephalopathy (SE) is a rare prion disorder characterized by progressive cognitive dysfunction and mortality. Affected patients can observe a wide variety of neurological symptoms, such as myoclonus, dementia, cerebellar signs, and others. We present a case of laboratory-confirmed SE in an otherwise healthy 57-year-old medical professional who initially presented with nonspecific and unique "head in a fish-bowl" dissociation and cognitive decline. No social risk factors were ever identified other than his healthcare career, but subsequent neuroimaging, serology, and lumbar puncture confirmed a diagnosis of sporadic SE due to unknown etiology. He was then treated symptomatically and referred ultimately to palliative care. The patient passed while in hospice care with time from the initial diagnosis to mortality being only 42 days. Given his vague but uniquely rapid deterioration and subsequent mortality, we highlight an opportunity to discuss diagnosis, management, quality improvement, and ethical concerns associated with SE prognosis. We aim to help primary care physicians and neurologists better elucidate the risk factors, signs and symptoms, and pathophysiology of SE to make an early diagnosis. Symptoms can then be managed effectively and palliative services coordinated via a legal and compassionate shared decision-making approach. We recommend that once a diagnosis is made, a discussion with the patient and their family about advance directives and end-of-life care be coordinated as soon as reasonably possible. This should be carried out by a multidisciplinary team consisting of the patient's primary care physician and neurologist, as well as a social worker, palliative care physician, and counselor (spiritual or otherwise). It is our hope that through a better understanding of these factors in SE care, quality of life improvement protocols in similarly-debilitating neurocognitive diseases can be developed.

## Introduction

Spongiform encephalopathies (SEs) are rare and uniformly fatal neurodegenerative diseases that are difficult to identify due to their sudden onset and rapid disease progression. Creutzfeldt-Jakob Disease (CJD), variant Creutzfeldt-Jakob Disease (vCJD), Gerstmann-Straussler-Scheinker Syndrome (GSSS), Fatal Familial Insomnia (FFI), and kuru are categories of currently identified human prion diseases [1]. The annual incidence of prion conditions was found to be around 1.2 million between 2003 and 2015 in the United States [2]. Most patients that present with CJD are over 60 years of age and about 80%-85% of cases are sporadic without an identifiable cause (sCJD). The rest of the cases are heritable or rarely due to iatrogenic contamination (1% of cases) [3].

The most serious complication of CJD is mortality, but patients also can present with a myriad of global and focal neuropsychiatric deficits. Patients may present with dementia, amnesia, myoclonus, visual disturbance, and cerebellar signs, among others. Survival depends on country and ethnicity, and while incidence and prevalence are low due to the rarity of the disease, prognosis and survival are very poor [2]. Chinese patients with sCJD have a median survival of 7.1 months with 78.5% one-year mortality, while European patients with sCJD have a median survival of five months with 85.8% one-year mortality [4]. Overall annual mortality from a survey of 3,720 sCJD cases from 1993-2002 in Canada, Australia, and Europe was 1.39 per million [3,4]. From 1979 to 2006, sCJD mortality in the United States was 1 per million [5]. 

Given the neurocognitive considerations and uniform mortality associated with SE, ethical and end-of-life (EOL) care decisions are of particular concern. We present a case of SE with a uniquely rapid course, highlighting the need for diagnosis and coordinating advanced directives as early as possible.

## Case presentation

We present here a highly educated 57-year-old Caucasian man who presented to his primary care provider with four weeks of general cognitive complaints, including working memory dysfunction, fatigue, and difficulty with attention. The patient denied any known inciting event, dietary or medication changes, trauma, falls, or new acute psychological stressors. He reported an anal fistulectomy procedure performed four months prior. He worked in healthcare and was married with children but had no other pertinent social or personal medical history. There was no known history of autoimmune disease, migraine, stroke, or seizure disorder. Family medical history was positive for hemochromatosis, hypertension, and coronary artery disease in his father.

During the initial evaluation, the patient noted a number of executive dysfunctions, including difficulty with short-term memory (e.g. forgetting destination mid-travel), focus (e.g. inability to multi-task), and motor deficits (e.g. right arm rigidity and weakness, aberrant extremity movements, and slight ataxia). While golfing a week prior, he reported a feeling of visual and general dissociation, describing a feeling of “full disconnect from his upper extremities” and a “fish-bowl” sensation where he felt as though it was not him hitting the ball. The day before evaluation, while playing ping pong at his home, he stated he was having difficulty tracking the ball on the right side and missing the ball more frequently than was his baseline. His wife confirmed that she had noticed overall changes in his personality and that his affect appeared flatter than normal. He had occasional neck pain and stiffness with radiation to the right arm but denied overt headache, loss of consciousness, focal weakness, or paresthesias. He was originally concerned that the complaints were a product of his job, which resulted in him fearing performing his duties at work and ultimately led to a reduction in hours. However, when his symptoms persisted, the patient felt it necessary to present to his primary care physician (PCP).

His PCP ordered a complete blood count, comprehensive metabolic panel, thyroid-stimulating hormone, folate, cyanocobalamin, and ferritin, which were all unremarkable. His Mini-Mental Status Examination (MMSE) showed an alert and oriented patient to person, place, and time, normal mood and mildly restricted affect, ability to speak in full sentences intelligibly, grossly intact cranial nerves II-XII, and no focal neurologic deficits. The patient was then referred to Infectious Disease and our Neurology team for further evaluation. His only findings of concern on the physical exam were a Saint Louis University Mental Status (SLUMS) score of 24/30 [6], losing points on questions 6, 7, 8, and 11. Considering his age and level of education, he qualified for a preliminary diagnosis of mild neurocognitive disorder (MNCD). The differential diagnosis at this point included infectious, metabolic, or autoimmune etiologies.

Due to the patient’s subjective history upon examination of progressive subacute complaints, additional testing was warranted. He underwent further blood work, neuroimaging, and cerebrospinal fluid (CSF) testing. Hematologic testing for Epstein-Barr virus (EBV) infection was positive (>600.0 U/mL). With regard to CSF, his glucose, red and white blood counts, myelin basic protein, venereal disease research laboratory (VDRL), *Borrelia burgdorferi* titer, oligoclonal IgG bands, neuron-specific enolase, xanthochromia, West Nile virus, herpes simplex virus, fluid gram stain, and EBV were all unremarkable. Total protein was found to be elevated (80 mg/dL) along with T-tau protein (2194 pg/mL). Qualitative testing for RT-QuIC and 14-3-3 protein were both positive. A second evaluation by another Neurologist one week later showed a MOCA score of 27/30, with 2/5 recall. Two weeks later, the patient then presented with a deteriorating neurocognitive picture with a MOCA score of 21/30, vertical nystagmus and visuospatial deficits, rare myoclonus, wide-based gait, and perceived difficulty recognizing space around him. 

**Figure 1:**
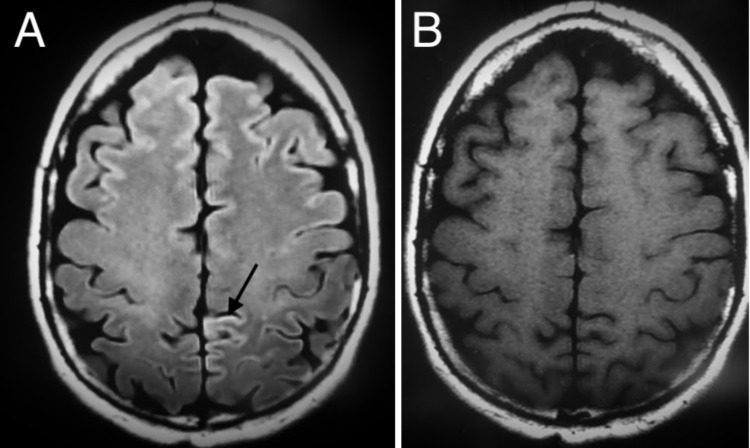
MR image of the brain with and without contrast (A) T2 FLAIR sequence transverse view shows an area of signal intensity along the paramedian portion of the left parietal lobe consistent with cortical ribboning in comparison to (B) T1 sequence (arrow).

Magnetic resonance imaging (MRI) of the brain with and without contrast T2 fluid-attenuated inversion recovery (FLAIR) sequence showed increased signal intensity along the paramedian portion of the left parietal lobe, consistent with cortical ribboning. There were similar areas of concern in the bilateral frontal lobes. No brain parenchymal abnormality, hydrocephalus, mass effect, hemorrhage, or midline shift were evident. MRI of the cervical spine was also taken that showed mild multilevel degenerative changes but was otherwise unremarkable (Figure 1).

**Figure 2:**
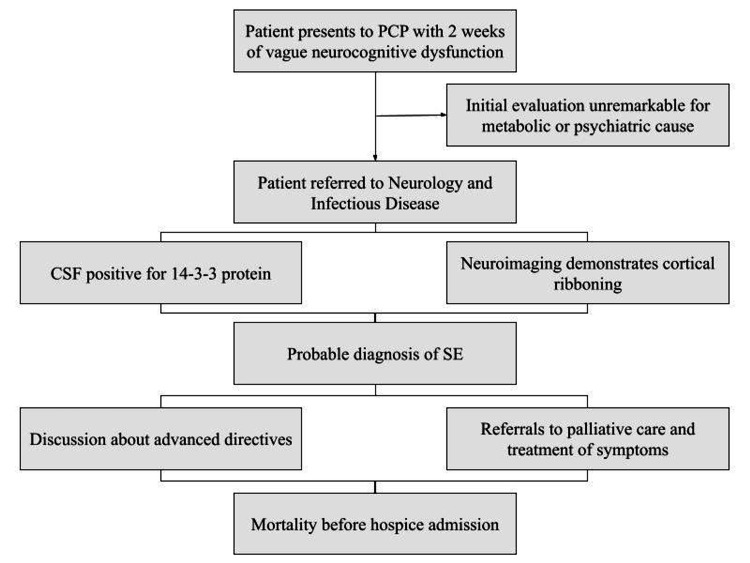
Patient care episode timeline. PCP: primary care physician; CSF: cerebrospinal fluid; SE: spongiform encephalopathy.

An electroencephalogram (EEG) was considered but deemed unnecessary considering the patient's prognosis and a probable diagnosis of sporadic SE of undetermined etiology was made. Upon follow-up with the family, it was noted that in the one month since the initial presentation to his PCP, the patient had become increasingly forgetful, ataxic, and developed profound personality changes. Considering the patient’s deteriorating picture and lack of disease-modifying treatments, therapy centered on palliative measures and symptom control. A fluoroscopic epidural blood patch was placed for post-dural lumbar puncture headache and a home infusion of methylprednisolone for five days was prescribed along with omeprazole for gastric reflux prophylaxis. Trials of gabapentin or acetylcholinesterase inhibitor were considered but were refused by the family due to the aggressive nature of the patient’s course, the lack of supportive evidence for these drugs for SE, and the family's desire to prolong the patient's functionality. Palliatives measures included referrals to physical and occupational therapy, functional medicine, physical medicine and rehabilitation, and home healthcare. The patient, deemed to be of sound decision-making capacity but at risk of falling below this clinical threshold in the near future, was encouraged to finalize advanced directives with his family. The patient was ultimately referred to hospice for EOL care but passed away before arrangements could be made or further empiric treatments attempted to increase survival or improve symptoms. The time from the initial diagnosis to mortality was 42 days. Post-mortem neuropathologic diagnosis was not available (Figure 2).

## Discussion

SE is a very rare disorder characterized by progressive neurocognitive deterioration with a dismal prognosis. Common neuropsychological morbidity includes apraxia, amnesia, and impaired attention [7]. As a result, therapy is aimed at palliation rather than curative intent [2,7]. Similarly, there are major ethical and EOL concerns that may complicate a patient’s course, their relationship with their family, and complicate navigation of the healthcare system. We present a case of uniquely rapid neurocognitive decline and mortality due to SE and use the clinical lessons learned to pontificate on best practices in EOL care and address the ongoing issue of delayed hospice admission. 

SE is diagnosed based on Centers for Disease Control and Prevention (CDC) guidelines, which include definite and probable diagnoses [2]. A definite diagnosis can only be determined by detection of protease-resistant PrP or scrapie-associated fibrils by neuropathology, immunocytochemistry, or western blot [2]. A probable diagnosis can be made with two out of four clinical features along with at least one positive laboratory test. Clinical features include myoclonus, visual or cerebellar signs, pyramidal/extrapyramidal signs, or akinetic mutism. Suggestive laboratory results are the presence of an atypical EEG with periodic sharp wave complexes, a positive 14-3-3 assay with disease duration of fewer than two years, or the presence of a high signal at the caudate or putamen on MRI or at least two cortical areas on diffuse-weighted imaging (DWI) or FLAIR MRI [2]. In this patient, a probable diagnosis was made based on his myoclonus, visual dysfunction, SLUMS downtrend, cortical ribboning on neuroimaging, and positive 14-3-3 protein on CSF testing. Alternative diagnoses that were considered included EBV encephalitis, neurosyphilis, primary dementia, Parkinson’s disease, central nervous system (CNS) lymphoma, and vascular dementia [8-11]. However, given the patient’s age, signs and symptoms, neuroimaging, and laboratory testing, each of these differentials were highly unlikely compared to SE [7,10]. Neuropathologic examination post-mortem would have provided the definitive diagnosis after-the-fact.

The patient was highly educated and worked in the healthcare field, so had better-than-average access to services and specialists. As is the case with many subacute and nonspecific patient presentations, historically, it can be difficult to parse out who the patient should be seen by or referred to at the time of initial presentation. Fortunately, his PCP made the intuitive decision to order a battery of hematologic lab tests and refer the patient to Neurology for further evaluation. For patients with leaner socioeconomic and healthcare resources, the initial evaluation by an Emergency Medicine or primary care physician is even more important. Hematologic testing and neuroimaging coupled with strong follow-up and continuity of care will help to make the diagnosis quicker and assuage symptoms more effectively. 

The ethical considerations that must be addressed in this case, as well as other causes of rapidly progressive neurocognitive deterioration, pertain to beneficence and autonomy. Once a diagnosis is made, it is imperative to begin transitioning the patient to end-of-life care [12]. This should include symptom management, assessing the possible need for hospice care now or in the future, as well as completion of an advanced directive and other aspects of end-of-life decision making [12-14]. However, limitations that preclude this have been found and set the stage for roadblocks that practitioners can address. These include the 11th-grade reading level in which it is written, which is three grades above the national average, its complex medical and legal wording within the documents, as well as deficient training in residency for having EOL conversations [15,16]. Tactics that practitioners can use to achieve better EOL care and efficient hospice admission include starting the advance directive process earlier, coordinating conversations person-to-person to allow for effective communication, and instituting staff protocols to assist the patient in navigating legal documentation. Additionally, candid discussions can ensure clarification on what the patient wants and limit an inaccurate interpretation of what a “quality” life means to them. Ultimately, better clinical care would likely follow [14,17-18].

Given that most physicians will encounter palliative care and EOL scenarios in their careers, formal education in these domains should improve. Better training in medical school or residency may have the greatest impact, but continuing medical education (CME) for practicing physicians also presents an opportunity [16,19]. Although the coronavirus disease-19 (COVID-19) pandemic has led to an increase in advance directive online course completions, there is an opportunity to improve training among young physicians in the sensitive and complex topics of patient autonomy and beneficence [20]. For example, the Accreditation Council of Graduate Medical Education (ACGME) could integrate these principles into the curricula of residency programs. Similarly, neurologists should especially be competent in EOL discussions, where its value would manifest predominantly in cases of swift and progressive neurocognitive deterioration, such as the patient in this report.

Our patient's rapid demise precluded hospice admission, but in general, this should be initiated as soon as possible given logistic health system challenges. This is especially true for patients of lesser socioeconomic status and with potentially fewer palliative options. Subsequently, experimental treatments may be trialed, but an effective multidisciplinary focus on ethical EOL care also allows an opportunity for the patient and his or her family to have more support during the course of the disease. Patient autonomy and beneficent care are thus preserved in a fragile, taboo, and emotional period. 

This study is limited by its nature as a case report. Future studies should characterize ethical and palliative care protocols for a thorough shared decision-making approach to care for physicians and their patients.

## Conclusions

Spongiform encephalopathy’s rarity, morbidity, and mortality make it a difficult disorder to study and to coordinate end-of-life care. This case helps to highlight an interesting presentation of subacute and progressive neurocognitive dysfunction and uniquely rapid demise in a relatively young and otherwise healthy individual without risk factors. The authors hope to add to the growing body of literature aimed to answer questions about spongiform encephalopathy, in order to help neurologists and primary care clinicians make confident steps toward diagnosis, note the need for better treatment protocols, and promote discussion of ethical end-of-life care among similar neurocognitive disorders.
